# Is sex necessary?

**DOI:** 10.1186/1741-7007-9-56

**Published:** 2011-08-31

**Authors:** Sheng Sun, Joseph Heitman

**Affiliations:** 1 Department of Molecular Genetics and Microbiology, Duke University Medical Center, Durham, NC 27710, USA

## Abstract

Fungal sexual reproductive modes have markedly high diversity and plasticity, and asexual species have been hypothesized to arise frequently from sexual fungal species. A recent study on the red yeasts provides further support for the notion that sexual ancestors may give rise to shorter-lived asexual species. However, presumed asexual species may also be cryptically sexual, as revealed by other recent studies.

See research article: http://www.biomedcentral.com/1471-2148/11/249

## Commentary

For most fungal species, unlike most animal species, sex is not obligatory. Although a few fungi may be obligately sexual, most can reproduce either sexually or asexually, and a significant minority are classified as exclusively asexual. Since even among closely related species, such as those of the *Candida *species complex [1,2], sexual species are interspersed with those for which no sexual reproduction has been documented, it has been suggested that as well as switching on and off within a species, sexual reproduction may be readily lost during evolution. An alternative explanation is that sex in apparently asexual species is not absent but cryptic. Because the pathogenicity of some fungal species depends on their ability to switch to sexual reproduction in their multicellular host, the question of sex in fungi is of more than academic interest. Attempts to answer it, including one just published in *BMC Evolutionary Biology *[3], have focused on the quest for evidence for the existence of genes required for sexual reproduction in asexual fungi, and for selective pressure acting on those genes.

**Figure 1:**
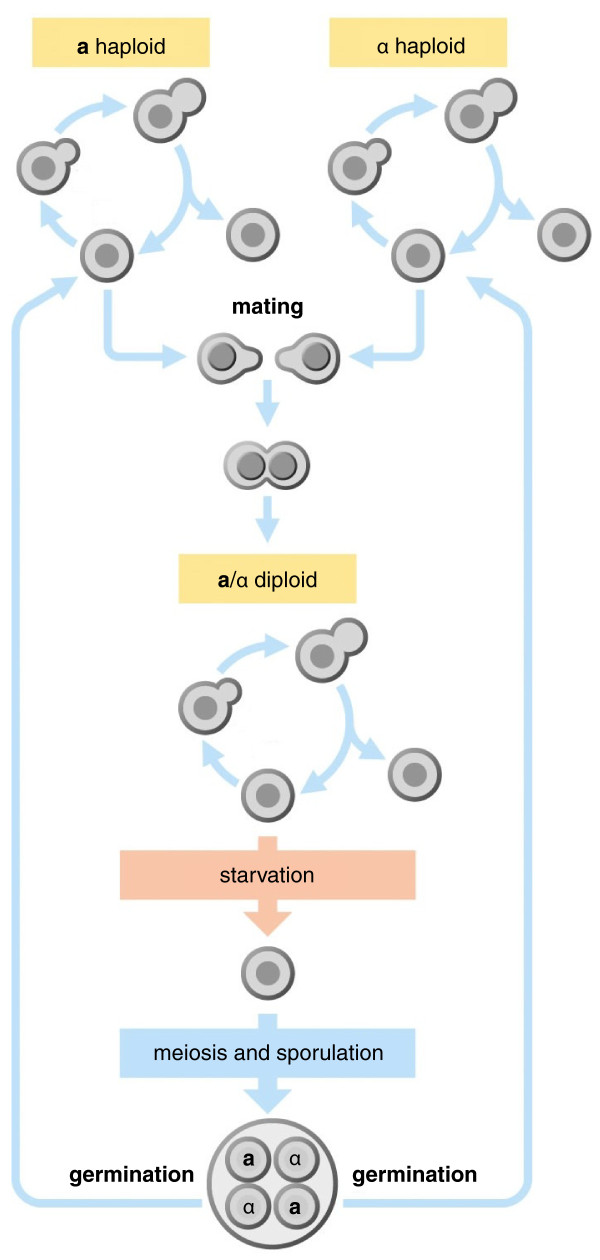
**The life cycle of a unicellular yeast**. The budding yeast *Saccharomyces cerevisiae *is an example of a sexual unicellular yeast, with haploid cells of two mating types, **a **and α, that mate when pheromones produced by each cell is recognized by pheromone receptors expressed on the opposite cell type: this stimulates fusion of the two cells to produce an **a**/α diploid cell that grows vegetatively when nutrients are plentiful, but upon starvation undergoes meiosis to produce four haploid spores that can remain dormant until conditions improve. Reproduced with permission from Figure 2-5 of Morgan DO, *The Cell Cycle*, Oxford University Press (2007).

Sexual reproduction entails, at a minimum, the fusion of two haploid cells to form a diploid cell that subsequently undergoes meiosis to produce four haploid progeny. In fungi, sex is defined by a locus, the mating type or *MAT *locus, that contains genes encoding cell identity determinants. In the most familiar case (that of *Saccharomyces cerevisiae*), homoeodomain and alpha-domain transcriptional regulators control genes required for sexual reproduction, including those encoding pheromones and pheromone receptors whereby haploid fungal cells signal to one another to initiate fusion. The apparatus of meiosis is also of course required. In this model budding yeast, there are two mating types, known as **a **and α, and fusion can occur only between cells of opposite mating type (Figure 1). But there are many variants of this system: in some species, mating can occur between cells of the same mating type; and in others, there can even be multitudes of different mating types.

## It is easier to lose sexual reproduction than to regain it

In principle, an asexual life cycle could arise from a sexual one by just one nucleotide substitution in a key gene located within *MAT*, or otherwise involved in mating or meiosis. The perturbations to sexual mechanisms that have actually been observed include non-functionalization of the homeodomain (HD) or pheromone/pheromone receptor (P/R) genes (either by gene deletions, point mutations, improper splicing, or changes in regulation that render these genes no longer functional in mating), or chromosomal rearrangements (translocations of the HD or P/R genes, inter-chromosomal recombination or fusions resulting in different configurations of the *MAT *loci) [1,2,4-7].

By contrast, once a species has become asexual it is unlikely to regain sexual reproduction, both because reversion that specifically corrects a defect is a relatively rare event, and because selection on *MAT *and meiosis genes would have been relaxed while the organism was reproducing asexually, allowing further deleterious mutations to accumulate. Restoration of sex in asexual lineages would thus be likely to require co-occurrence of back mutations in multiple genes, which would happen at extremely low frequency. Only one example of such a rare restoration of sexual reproduction from asexual lineages has been suggested in a parthenogenetic mite that seems to have re-evolved sexual reproduction [8].

One parsimonious explanation for the frequent co-occurrence in closely related unicellular fungal species of sexual and asexual strains, therefore, is that sexual reproduction could be frequently lost; indeed, sexual reproduction has been seen to be readily lost under laboratory conditions in some fungi (for example, [9]), and several recent studies have suggested that asexual fungi arise frequently in nature ([3] and references therein). Population genetic studies of many asexual species, for example, have found genomic signatures of recombination, possibly indicating a sexual history, since recombination occurs frequently during mating and meiosis.

## Asexual reproduction has more advantages for fungi than for multicellular organisms

It is widely thought that the asexual strains found in nature are evolutionary dead-ends derived from sexual species, and that these die out after a relatively short time, the long-term advantages of sex outweighing any short-term advantages of the asexual life cycle. This balance, however, is difficult to gauge for fungi. The main advantages of sex are the possibility of greater genetic diversity and the benefits of sporulation, which is associated with sexual reproduction in fungi and occurs in adverse conditions, allowing the fungus to persist in protected and dormant form as spores until conditions improve, or to disperse to a more favorable environment.

**Figure 2:**
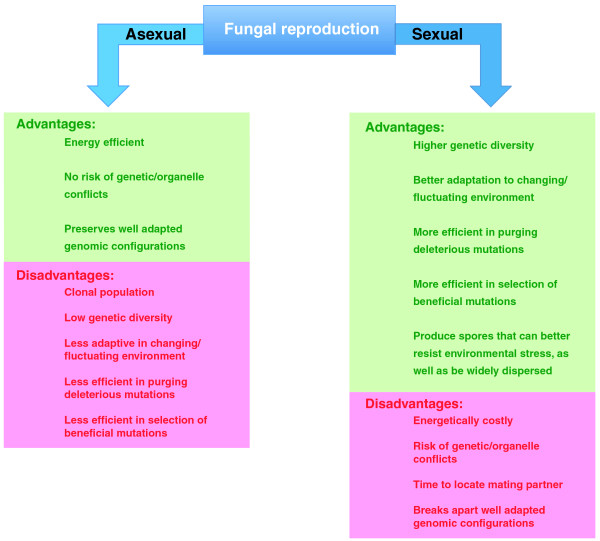
**Advantages and disadvantages of a sexual or an asexual life cycle**.

However, sex also has costs [10], including the metabolic cost of maintaining the mating system and of mating itself, and reassortment of well adapted gene combinations (recombination cannot be guaranteed to produce favorable new combinations of genes); and at least in the short term, an asexual strain may out-compete a sexual one in vegetative growth (Figure 2). It can even be argued that the consequences of sexual reproduction, such as the generation of aneuploidy, may provide progeny with enhanced potential for evolving a selective advantage over the parent sexual strain. In fungal species that undergo sexual reproduction, aneuploid progeny - that is, progeny with duplicated or missing chromosomes - can be produced at a relatively high frequency (for example, [2]). Aneuploidy at the *MAT *locus and/or at genes involved in mating or meiosis could impair mating ability, and relax the selection pressure on these components, allowing degeneration of these genes and, if the strains with impaired *MAT *or meiotic genes mate with the normal population, perhaps the production of aneuploid progeny at even higher frequency. The relaxed selective pressure on the duplicated genes could result in higher phenotypic diversity among these progeny, and aneuploidy itself also increases phenotypic diversity by virtue of genes overexpressed as a result of altered dosage, and thus there is a greater chance that a 'fitter' asexual progeny might arise and outcompete the parental strain. The potential for diversification could also increase the potential for evolution of strains that can directly inhibit the growth of other strains, as with the 'killer' effect observed in some yeast species [5,11].

Because microbial species such as unicellular yeasts can rapidly adapt to environmental changes, or to diverse environmental niches, asexual species may readily avoid direct competition with the original sexual lineage.

All of these are reasons for expecting asexual species of unicellular yeasts to persist more effectively than might be expected for multicellular animals, in which asexual species are extremely rare. Moreover, efficient selection against asexuality requires a large population. We currently know very little about the effective population size of most fungal species. But a small effective population size could allow an evolved asexual lineage to survive and persist through random genetic drift; this could occur even in a large population if the population is divided into isolated subpopulations in which sexual reproduction is frequent within each subpopulation but uncommon between subpopulations. And just this sort of mating preference has been observed to evolve under laboratory conditions in budding yeast [12].

## Cryptic sex

Although many fungi are currently classified as asexual, it is possible that laboratory conditions conducive to mating remain to be defined, or compatible mating partners have not as yet been isolated from natural environments for these species. An example is the recent discovery of the sexual cycle of the fungal pathogen *Aspergillus fumigatus*, which requires six months incubation under specialized conditions in the dark [13]. It is also possible that the same fungus can engage in different modes of reproduction (sexual and asexual) in different locations (for example, when free-living or in a multicellular host) or at different times (for example, sexual reproduction of some plant pathogens is coincident with specific developmental stages of their hosts). To fully understand the mating behavior of a fungus requires extensive environmental sampling and laboratory mating assays.

Detailed genomic and genetic studies can also provide clues as to whether species may be sexual. Whole genome analyses of many putatively asexual fungi have often revealed the presence of *MAT *loci and suites of apparently intact mating and meiotic genes. In their survey of the red yeast Sporidiobolales, for example, Coelho *et al*. [3] found retention of apparently functional copies of the genes encoding the homeodomain mating-type transcriptional regulators and the pheromone receptors in several of the 16 asexual species investigated. If sexuality has been lost in these species, the loss must have been very recent to avoid the erosive signature of gene loss and decay. Population genetic studies have often revealed that presumptive asexual fungi exist in a 1:1 ratio of opposite mating types and with evidence of recent recombination, possibly reflecting cryptic sexuality.

One question facing the approach of interpreting the reproductive mode through genome analysis is: what would the genome of an asexual fungus look like? Coelho *et al*. [3] found several asexual species of red yeasts in which the mating-type genes encoding the homeodomain transcription factors are disabled by mutations, and infer that these mutations are a cause, or a consequence, of asexuality. Yet *Lodderomyces elongisporus*, which is classified as a self-fertile sexual fungus (it makes an ascus with a single lone spore), has no discernible *MAT *locus (it lacks the **a**1, **a**2, α1, and α2 genes), and is also missing the **a **factor pheromone, the **a **pheromone receptor, and the **a **pheromone transporter genes required for signaling in mating [1]. Is it truly sexual, or does it produce one-spored asci asexually? *Candida parapsilosis *is an **a**/**a **diploid for which no mating has been observed; it has also lost the **a1 **HD transcription factor and lacks a key region of the α-factor receptor involved in response of *Candida albicans *white cells to pheromone; additionally, no isolate of the α mating type has yet been isolated from nature or patients [1,7]. Thus, *C. parapsilosis *could be asexual or it may undergo cryptic unisexual mating like *C. albicans *and *Cryptococcus neoformans *[14,15]. Or rare α isolates may remain to be discovered. *Candida glabrata *is a haploid yeast related to *S. cerevisiae*, and natural populations contain both **a **and α isolates; however, no mating has yet been detected in the lab despite prodigious efforts [5]. *C. glabrata *could be asexual, or it may simply be that its mating rituals will require further wile on our part to discern patterns of cryptic sexuality. Finally, a note of caution. *Candida lusitaniae *has lost the α2 HD gene at *MAT*, and *Candida guilliermondii *has lost both the **a**1 and α2 genes, yet both have maintained complete sexual cycles [1,2]. Thus, loss of *MAT *genes is not synonymous with asexuality.

Given the flexible reproduction modes and enormous diversities of phenotypic traits and environmental niches of fungi, as well as their complicated population dynamics, studies of their reproductive and mating systems are surely going to provide us with more surprises.

## References

[B1] Butler G Rasmussen MD Lin MF Santos MA Sakthikumar S Munro CA Rheinbay E Grabherr M Forche A Reedy JL Agrafioti I Arnaud MB Bates S Brown AJ Brunke S Costanzo MC Fitzpatrick DA de Groot PW Harris D Hoyer LL Hube B Klis FM Kodira C Lennard N Logue ME Martin R Neiman AM Nikolaou E Quail MA Quinn J Evolution of pathogenicity and sexual reproduction in eight *Candida *genomes Nature 2009 459 657 662 10.1038/nature08064 19465905 PMC2834264

[B2] Reedy JL Floyd AM Heitman J Mechanistic plasticity of sexual reproduction and meiosis in the *Candida *pathogenic species complex Curr Biol 2009 19 891 899 10.1016/j.cub.2009.04.058 19446455 PMC2788334

[B3] Coelho MA Goncalves P Sampaio JP Evidence for maintenance of sex determinants but not sexual stages in red yeasts, a group of early diverged basidiomycetes BMC Evol Biol 2011 11 249 10.1186/1471-2148-11-249 PMC3236058 21880139

[B4] Coelho MA Rosa A Rodrigues N Fonseca A Goncalves P Identification of mating type genes in the bipolar basidiomycetous yeast *Rhodosporidium toruloides*: first insight into the *MAT *locus structure of the *Sporidiobolales* Eukaryot Cell 2008 7 1053 1061 10.1128/EC.00025-08 18408057 PMC2446649

[B5] Muller H Hennequin C Gallaud J Dujon B Fairhead C The asexual yeast *Candida glabrata *maintains distinct a and α haploid mating types Eukaryot Cell 2008 7 848 858 10.1128/EC.00456-07 18375614 PMC2394967

[B6] Coelho MA Sampaio JP Goncalves P A deviation from the bipolar-tetrapolar mating paradigm in an early diverged basidiomycete PLoS Genet 2010 6 e1001052 10.1371/journal.pgen.1001052 20700437 PMC2916851

[B7] Sai S Holland LM McGee CF Lynch DB Butler G Evolution of mating within the *Candida parapsilosis *species group Eukaryot Cell 2011 10 578 587 10.1128/EC.00276-10 21335529 PMC3127640

[B8] Domes K Norton RA Maraun M Scheu S Reevolution of sexuality breaks Dollo's law Proc Natl Acad Sci USA 2007 104 7139 7144 10.1073/pnas.0700034104 17438282 PMC1855408

[B9] Xu J Estimating the spontaneous mutation rate of loss of sex in the human pathogenic fungus *Cryptococcus neoformans* Genetics 2002 162 1157 1167 12454063 10.1093/genetics/162.3.1157 PMC1462337

[B10] Xu J Cost of interacting with sexual partners in a facultative sexual microbe Genetics 2005 171 1597 1604 10.1534/genetics.105.045302 15998718 PMC1456087

[B11] Marquina D Santos A Peinado JM Biology of killer yeasts Int Microbiol 2002 5 65 71 10.1007/s10123-002-0066-z 12180782

[B12] Leu JY Murray AW Experimental evolution of mating discrimination in budding yeast Curr Biol 2006 16 280 286 10.1016/j.cub.2005.12.028 16461281

[B13] O'Gorman CM Fuller HT Dyer PS Discovery of a sexual cycle in the opportunistic fungal pathogen *Aspergillus fumigatus* Nature 2009 457 471 474 10.1038/nature07528 19043401

[B14] Lin X Hull CM Heitman J Sexual reproduction between partners of the same mating type in *Cryptococcus neoformans* Nature 2005 434 1017 1021 10.1038/nature03448 15846346

[B15] Alby K Schaefer D Bennett RJ Homothallic and heterothallic mating in the opportunistic pathogen *Candida albicans* Nature 2009 460 890 893 10.1038/nature08252 19675652 PMC2866515

